# Prelamin A Accumulation Attenuates Rac1 Activity and Increases the Intrinsic Migrational Persistence of Aged Vascular Smooth Muscle Cells

**DOI:** 10.3390/cells5040041

**Published:** 2016-11-15

**Authors:** Lauren J. Porter, Mark R. Holt, Daniel Soong, Catherine M. Shanahan, Derek T. Warren

**Affiliations:** 1 British Heart Foundation Centre of Research Excellence, Cardiovascular Division, King’s College London, London SE5 9NU, UK; laurenjadeporter@gmail.com (L.J.P.); dsoong@staffmail.ed.ac.uk (D.S.); cathy.shanahan@kcl.ac.uk (C.M.S.); 2 Randall Division of Cell and Molecular Biophysics, New Hunt’s House, King’s College London, London SE1 1UL, UK; mark_robert.holt@kcl.ac.uk; 3 MRC Centre for Reproductive Health, Queen’s Medical Research Institute, University of Edinburgh, Edinburgh EH16 4TJ, UK; 4 School of Pharmacy, University of East Anglia, Norwich Research Park, Norwich NR4 7TJ, UK

**Keywords:** prelamin A, migratory persistence, Rac1

## Abstract

Vascular smooth muscle cell (VSMC) motility is essential during both physiological and pathological vessel remodeling. Although ageing has emerged as a major risk factor in the development of cardiovascular disease, our understanding of the impact of ageing on VSMC motility remains limited. Prelamin A accumulation is known to drive VSMC ageing and we show that presenescent VSMCs, that have accumulated prelamin A, display increased focal adhesion dynamics, augmented migrational velocity/persistence and attenuated Rac1 activity. Importantly, prelamin A accumulation in proliferative VSMCs, induced by depletion of the prelamin A processing enzyme FACE1, recapitulated the focal adhesion, migrational persistence and Rac1 phenotypes observed in presenescent VSMCs. Moreover, lamin A/C-depleted VSMCs also display reduced Rac1 activity, suggesting that prelamin A influences Rac1 activity by interfering with lamin A/C function at the nuclear envelope. Taken together, these data demonstrate that lamin A/C maintains Rac1 activity in VSMCs and prelamin A disrupts lamin A/C function to reduce Rac1 activity and induce migrational persistence during VSMC ageing.

## 1. Introduction

Ageing is the greatest risk factor in the development of cardiovascular disease yet the mechanisms underlying vessel ageing and how ageing impinges on vascular cell function remain poorly understood [1,2]. Vascular smooth muscle cells (VSMCs) are the major cell type of the arterial wall and normally exist in a contractile, differentiated state to maintain vascular tone. However, contractile VSMCs are not terminally differentiated and retain the ability to dedifferentiate to a proliferative, migratory phenotype and enhanced VSMC motility is observed during development, vessel repair and in adverse vessel remodeling associated with restenosis and atherosclerosis [3,4,5]. VSMC phenotypic transition involves dramatic actin remodeling which is regulated by Rho GTPase signalling pathways [6,7]. Recent evidence demonstrates that Rac1 is essential for VSMC migration and neointimal formation in vivo [8]. Moreover, Rac1 signalling is critical for switching between random and directionally persistent migration in a variety of cell types [9], although whether Rac1 performs this function in VSMCs remains unknown.

The nuclear lamina, a filamentous meshwork of A- (lamin A/C) and B- (lamins B1 and B2) type lamins, has emerged as a regulator of cytoskeletal organisation and cell motility [10]. The A-type lamins are mechanically coupled to the cytoskeleton via association with the linker of the nucleoskeleton and cytoskeleton (LINC) complex, that spans the nuclear envelope (NE) via interactions between giant nesprin isoforms on the outer nuclear membrane (ONM) and the sad1 and UNC84 (SUN) domain containing proteins (SUN1 and SUN2) that span the inner nuclear membrane (INM) and directly bind lamin A/C in the nucleoplasm [11]. Importantly, this mechanical coupling permits transmission of biophysical signals between the cytoskeleton and lamins to regulate lamin A/C organisation, gene transcription and nuclear deformability [12,13]. Lamin A is synthesized from the precursor protein prelamin A that undergoes a series of post-translational modifications, including farnesylation and proteolytic cleavage by FACE1 before mature lamin A is incorporated into the nuclear lamina [14,15,16,17]. However, mutations that disrupt lamin A processing give rise to Hutchinson-Gilford progeria syndrome (HGPS), a severe early ageing disease where patients have a toxic accumulation of mutant prelamin A, progerin, that accelerates senescence by inducing nuclear stiffening and disrupting nuclear integrity [18,19,20,21,22]. HGPS patients develop premature atherosclerosis due to VSMC dysfunction and usually die of myocardial infarction or stroke in their second decade of life [23,24,25]. Importantly, normal VSMCs exhibit an age-related depletion in FACE1, leading to impaired prelamin A processing and subsequent prelamin A accumulation [26,27]. More recently, we have demonstrated that prelamin A accumulates prior to senescence, during a presenescent growth phase, further supporting a role for prelamin A in promoting VSMC ageing and senescence [28].

HGPS-derived fibroblasts display impaired cell motility, suggesting that coupling between the nuclear lamina and the cytoskeleton is critical for efficient cell migration [29,30]. In agreement, disruption of LINC complex integrity, either in Laminopathic patient derived fibroblasts or by the over expression of the dominant negative nesprin KASH domain, triggers altered cell morphology and attenuates cell motility [13,29,30]. We have previously shown that prelamin A accumulation induces VSMC presenescence, yet whether age associated changes in NE architecture impact on VSMC motility remains unknown [28]. Therefore, we investigated the impact of VSMC presenescence and prelamin A accumulation on the morphology and migratory capacity of VSMCs. VSMC morphology, focal adhesion (FA) organisation, Rac1 activity and motility were altered during in vitro VSMC ageing. Importantly, prelamin A accumulation in proliferative VSMCs mirrored the FA, Rac1 activity and motility changes observed in presenescent VSMCs, suggesting that the A-type lamins form a feedback loop to influence Rac1 signalling and regulate the intrinsic migrational persistence during VSMC ageing.

## 2. Materials and Methods

### 2.1. Cell Culture

VSMCs were isolated from medial explants of human aortic tissue as previously described [31]. The VSMCs used in this study were derived from a 35-year-old female (35F) and a 54-year-old male (54M). VSMCs were cultured in M199 medium containing 20% fetal bovine serum. VSMCs were serially passaged in vitro and collected at proliferative (passages ~6–10), presenescent (passages ~12–14) and senescent (passage ~15+) growth stages, as described previously [27]. All culture medium was complemented with 1% penicillin/streptomycin and 200 μM l-Glutamine. Dharmacon smart pool control and FACE-1 specific small interfering RNA (siRNA) oligonucleotides were transfected into cells with the use of HiPerfect transfection reagent (Qiagen, Hilden, Germany) and incubated for 72 h.

### 2.2. Immunofluorescence Microscopy

For immunostaining, cells were fixed in 4% paraformaldehyde, permeabilised in 0.5% Nonidet P-40 alternative in phosphate buffered saline (PBS) and blocked in 1% bovine serum albumin (BSA) in PBS for 1 h. Cells were incubated with prelamin A (Santa Cruz, Dallas, TX, USA) or vinculin (Sigma-Aldrich, St Louis, MO, USA) primary antibody in blocking solution overnight at 4 °C. After washing with PBS, cells were incubated in fluorescence-conjugated secondary antibody solution or Rhodamine phalloidin (Invitrogen) for 1 h before staining cell nuclei with 4′,6′-Diamidino-2-Pheylindoledihydrochloride (DAPI). Cells were washed and mounted onto slides using Mowiol/Dabco media. Images were captured using a Zeiss Axioplan 2 light microscope and Volocity software (Perkin Elmer, Waltham, MA, USA) was used to control image acquisition and measure morphological parameters (area and circularity) by fluorescence detection.

### 2.3. Glutathione–S-Transferase (GST) Pull-Down

P21-associated kinase (PAK) is an immediate downstream effector of stimulated Rac1 and was used in this experiment to assay Rac1 activity. BL-21s were transformed with GST-PAK-P21 Rho binding domain plasmid (Addgene #12217) and expression was induced by the addition of 1 mM Isopropyl β-d-1-thiogalactopyranoside for 2 h at 37 °C. Bacterial pellets were suspended in PBS containing protease inhibitors, sonicated and proteins solubilised in 1% Triton-X for 1 h at 4 °C. Following centrifugation, recombinant protein was purified by incubating supernatant with glutathione-sepharose beads for 30 min at room temperature GST-protein beads (50 μL of 50% slurry) were incubated with 200 μg VSMC lysate on a rotator for 2 h at 4 °C and washed three times. Bead-bound protein was eluted by boiling in β-mercaptoethanol sample buffer and Rac1 activity was analysed by sodium dodecyl sulphate-polyacrylamide gel electrophoresis (SDS-PAGE) and Western blot analysis.

### 2.4. Quantitative Polymerase Chain Reaction (qPCR) and Western Blot Analysis

FACE1 qPCR was performed as described previously [27]. For Western blotting, VSMCs were harvested into lysis buffer (10 mM Tris, pH 7.5, 150 mM NaCl, 1 mM EDTA, 1% Triton) supplemented with protease inhibitors. Cell lysates were sonicated and boiled in β-mercaptoethanol sample buffer before being subjected to SDS-PAGE and Western blot analysis using prelamin A (C-20), lamin A/C (N18) (Santa Cruz), emerin (Novus Biologicals., Oakville, ON, Canada), SUN2 (Abcam., Cambridge, UK), vinculin (Sigma., St Louis, MO, USA), nesprin-2 CH3 (described previously [32]) and Rac1 (Millipore., Watford, UK) antibodies.

### 2.5. Time-Lapse Video Microscopy and Migration Analysis

Cells were sparsely seeded in wells of a culture plate 24 h prior to filming. Time-lapse imaging was conducted using an Olympus IX81 microscope attached to a Hamamatsu Photonics Orca-R2 cooled CCD camera (Hamamatsu Photonics., Welwyn Garden City, UK). Phase contrast images were captured at varying positions using a motorised stage (LUDL Electronic Products, Hawthorne, NY, USA) every 5 min for 16 h. Individual cells were manually tracked throughout consecutive frames using an ImageJ software plugin (ImageJ1, National Institutes of Health (NIH), Bethesda, MD, USA). Using these x-y coordinates, migrational velocity and persistence were determined using Mathematica 6.0 (Wolfram Research Ltd., Witney, UK) custom-written notebooks kindly provided from Professor Graham Dunn and Daniel Soong, King’s College London.

### 2.6. Interference Reflection Microscopy (IRM)

Cells were cultured on glass-bottom culture dishes (MatTek Ltd., Ashland, MA, USA) for 18–24 h. IRM images were captured every 2 min using an incident light fluorescence attachment and a 63× oil immersion objective. Images were processed using ImageJ software in a method modified from previous studies [33]. Firstly, an FFT band pass filter was applied to generate binary images, making focal adhesions strikingly obvious against the background. Artifacts were removed manually by selecting and inverting to colour-match the background. The cell leading edge was selected and ImageJ measured the pixel count of individual focal adhesions. Composite images highlighted newly formed focal adhesions against original focal adhesions. The pixel count of newly formed adhesions was then expressed as a percentage of the total number of original pixels to give focal adhesion assembly (%).

### 2.7. Statistical Analysis

Data are presented as mean +/− SEM and p-values were calculated using paired (siRNA depletion) or unpaired (proliferative/presenescent) two-tailed Student’s t tests (Prism5, GraphPad Software Inc., La Jolla, CA, USA). One-way ANOVA with Bonferroni’s post-test were performed on proliferative/presenescent/senescent and sicontrol/siFACE1/silamins A/C data (GraphPad Prism software).

## 3. Results

### 3.1. Prelamin A Accumulation Drives Aged-Related Morphological Changes

**Figure 1:**
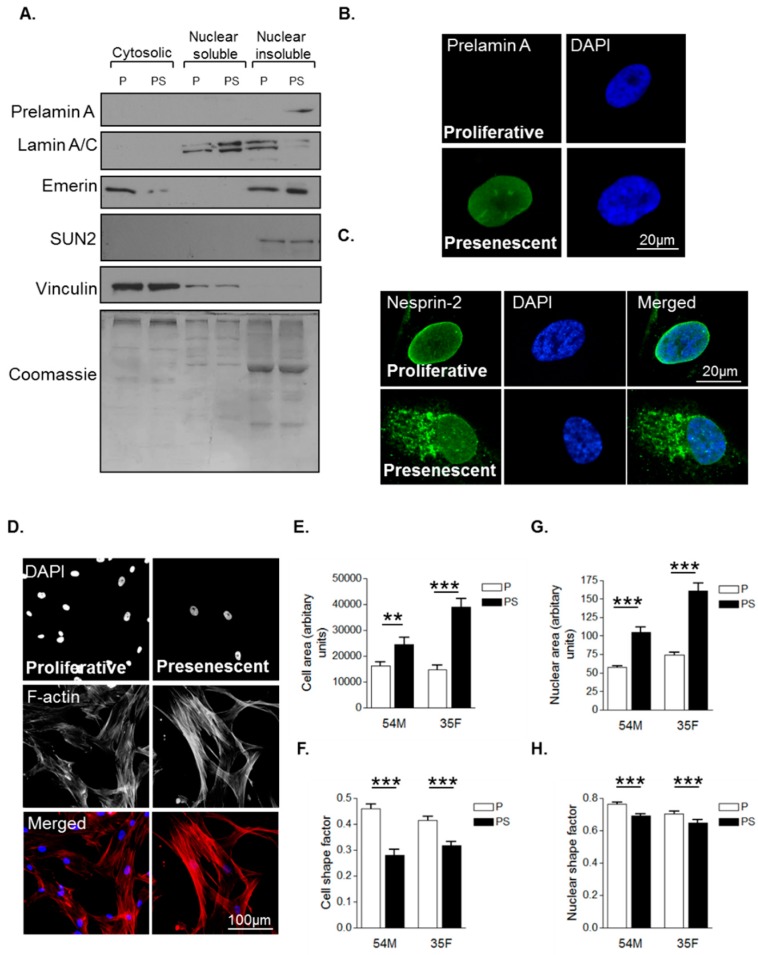
Characterisation of morphological changes during vascular smooth muscle cell (VSMC) ageing. (**A**) Western blot (WB) of cytoplasmic, nuclear soluble and nuclear insoluble fractionations of proliferative (P) and presenescent (PS) VSMCs; Representative confocal images of proliferative and presenescent VSMCs stained with (**B**) prelamin A (green) and 4’,6-diamidino-2-phenylindole (DAPI) (blue); (**C**) nesprin-2 CH3 (green) and DAPI (blue); (**D**) Rhodamine phalloidin (red) and DAPI (blue). Graphs show (**E**) cell area, (**F**) cell shape factor, (**G**) nuclear area and (H) nuclear shape factor of proliferative (P) and presenescent (PS) VSMCs based on the combined measurement of >300 VSMCs from two different isolates (54M, 35F) in three independent experiments (** *p* ≤ 0.01 and *** *p* ≤ 0.0001).

Primary human VSMCs were serially passaged in vitro to model VSMC ageing. As described previously, VSMCs have three distinct growth stages: an initial proliferative phase at early passage numbers when growth rate is high and prelamin A level is low; a presenescent phase when growth rate begins to subside and prelamin A accumulates; and finally, a senescent phase when proliferation terminates and prelamin A levels persist [27]. Western blot analysis (WB) confirmed that presenescent VSMCs possessed increased levels of prelamin A in the nuclear insoluble fraction compared to proliferative VSMCs (Figure 1A). Confocal immunofluorescence microscopy (IF) confirmed that the nuclei of presenescent VSMCs possessed increased prelamin A levels compared to their proliferative counterparts (Figure 1B). Next, we assessed the impact of presenescence on LINC complex integrity by performing IF using an antibody raised to the N-terminus of the nesprin-2 giant. IF revealed that nesprin-2 localised to the nuclear envelope of proliferative VSMCs, however, in presenescent VSMCs, nesprin-2 was mislocalised from the NE to the endoplasmic reticulum (Figure 1C). We next investigated the impact of presenescence on VSMC morphology by analysing 2-independent VSMC lines. Analysis revealed that presenescent VSMCs were larger and more elongated than their proliferative counterparts (Figure 1D–F) and possessed larger and more elongated nuclei (Figure 1G,H).

**Figure 2:**
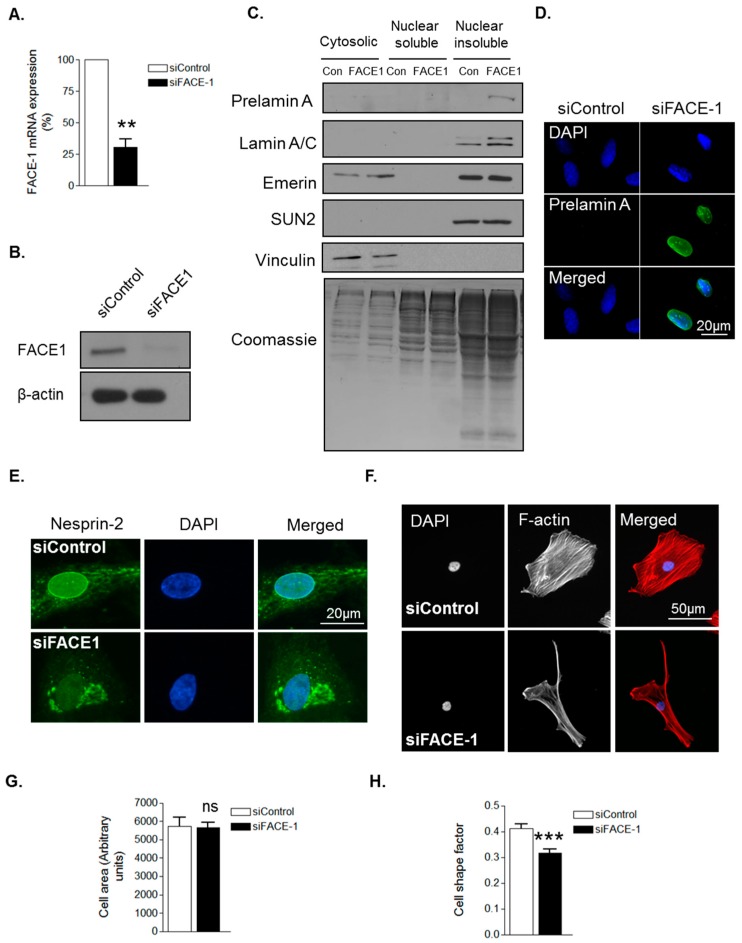
Prelamin A accumulation alters VSMC morphology. (**A**) Graph shows farnesylated proteins-converting enzyme 1 (FACE1) mRNA levels in control and FACE1-depleted VSMCs determined by qPCR and represents the combined data from three independent experiments (** *p* ≤ 0.01); (**B**) WB for FACE1 levels in control and FACE1-depleted VSMCs; (**C**) WB of cytoplasmic, nuclear soluble and nuclear insoluble fractionations of control and FACE1-depleted VSMCs; Representative confocal images of control and FACE1-depleted VSMCs stained with (**D**) prelamin A (green) and DAPI (blue); (**E**) nesprin-2 CH3 (green) and DAPI (blue); (**F**) Rhodamine phalloidin (red) and DAPI (blue). Graphs show (**G**) cell area; (**H**) cell shape factor of control and FACE1-depleted VSMCs based on >300 VSMCs from three independent experiments (*** *p* ≤ 0.0001).

To confirm that prelamin A influences VSMC morphology, we employed a FACE1 siRNA mediated knockdown approach. QPCR and WB confirmed efficient depletion of FACE1 mRNA and protein compared to controls (Figure 2A,B). Subcellular fractionation confirmed prelamin A accumulation in the nuclear insoluble fraction of FACE1-depleted VSMCs (Figure 2C) and IF analysis confirmed prelamin A accumulation in the nuclei of FACE1-depleted VSMCs (Figure 2D). Furthermore, IF also revealed that nesprin-2 was mislocalised from the NE in FACE1-depleted VSMCs (Figure 2E). IF analysis revealed FACE1-depleted VSMCs displayed no change in spread area but were more elongated than control cells (Figure 2F–H), suggesting that prelamin A contributes to age associated changes in VSMC morphology.

### 3.2. Prelamin A Accumulation Promotes Focal Adhesion Reorganisation

**Figure 3:**
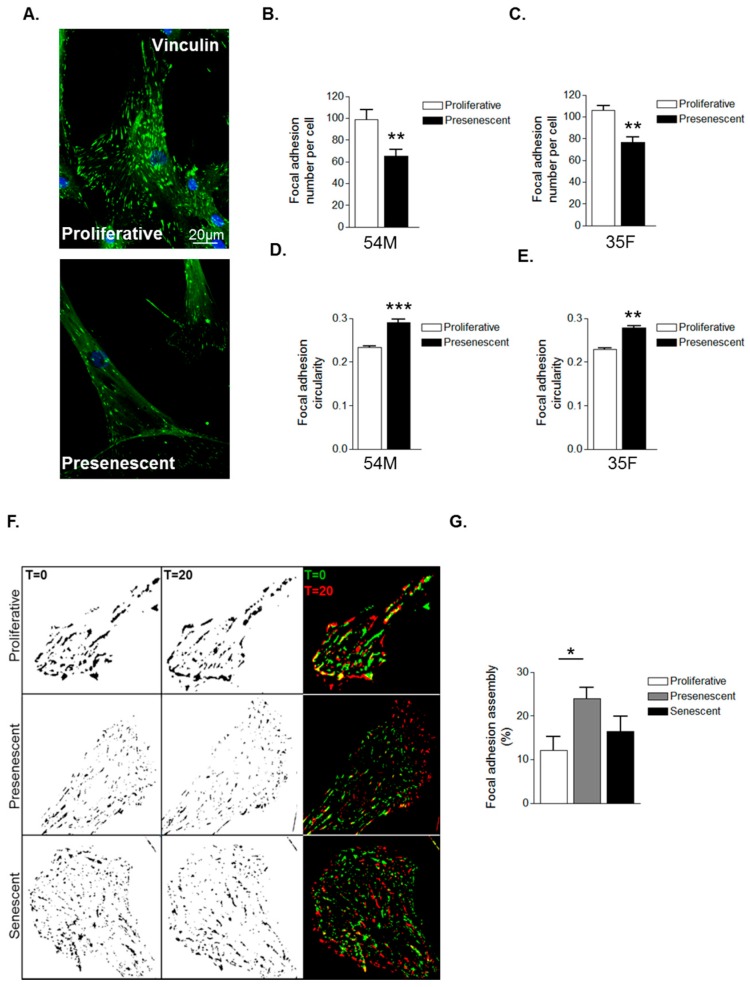
VSMC ageing influences focal adhesion organisation. (**A**) Representative confocal images of proliferative and presenescent VSMCs stained for vinculin (green) and DAPI (blue); Scale bar represents 20 μm. Graphs show (**B**,**C**) focal adhesion (FA) number per cell and (**D**,**E**) FA circularity of 54M and 35F VSMC isolates respectively. Data represent combined data analysing 500–1000 focal adhesions from >100 cells from three independent experiments (** *p* ≤ 0.01 and *** *p* ≤ 0.0001); (**F**) Representative IRM overlay images of proliferative, presenescent and senescent 35F VSMCs at t = 0 min (green) and t = 20 min (red); (**G**) Graph shows focal adhesion assembly over a 20-min period and represents the combined data of 15–20 cells per group pooled from three independent experiments (* *p* ≤ 0.05).

The above data suggest that prelamin A accumulation induces cytoskeletal reorganisation in VSMCs. As the actin cytoskeleton is tethered to the extracellular matrix (ECM) by focal adhesion complexes (FA), we next investigated whether prelamin A accumulation impacted on FA organisation. IF of vinculin stained VSMCs revealed FAs to be abundantly localised throughout proliferative VSMCs whereas presenescent VSMCs displayed fewer FAs that were redistributed to the cell periphery (Figure 3A–C). FAs were also more circular in presenescent compared with proliferative VSMCs (Figure 3D,E). Alterations in FA organisation reflect changes in FA dynamics, so we next performed interference reflection microscopy (IRM) to measure FA assembly. Presenescent VSMCs displayed enhanced FA assembly, indicating that presenescent VSMCs possess fewer, more dynamic FAs compared to proliferative and senescent VSMCs (Figure 3F,G).

**Figure 4:**
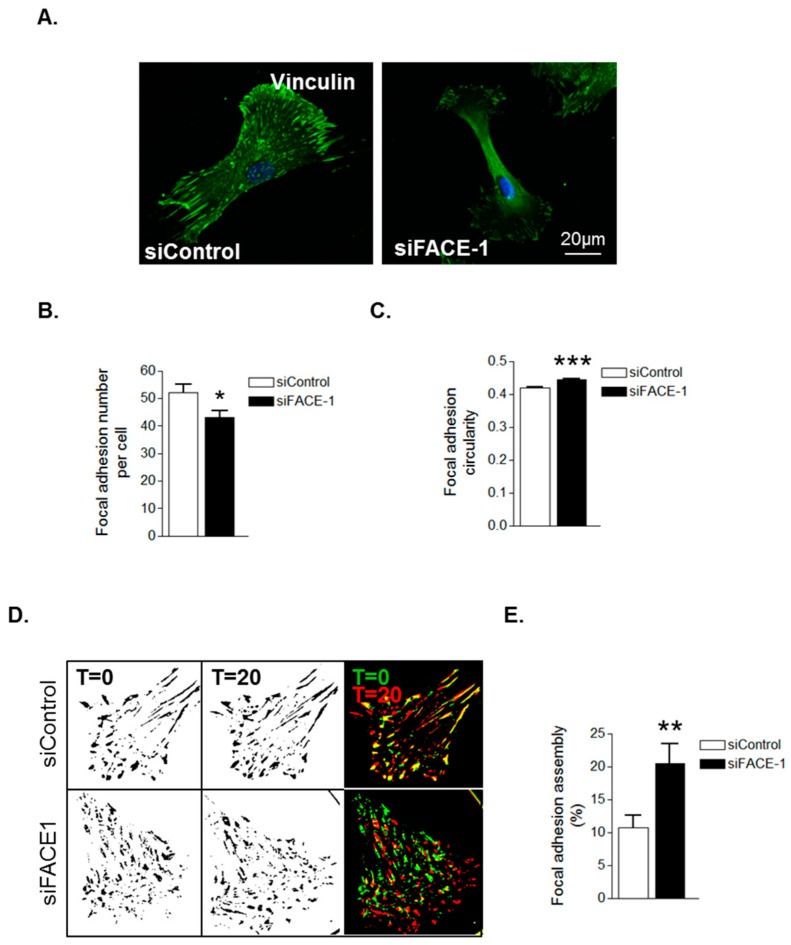
Prelamin A accumulation triggers FA reorganisation. (**A**) Representative confocal images of control and FACE1-depleted VSMCs stained for vinculin (green) and DAPI (blue). Scale bars represent 20 μm; Graphs show (**B**) FA number per cell and (**C**) FA circularity of control and FACE1-depleted VSMCs. Data represent combined data analysing more than 300 focal adhesions pooled from >50 cells per group from three independent experiments (* *p* ≤ 0.05 and *** *p* ≤ 0.0001); (**D**) Representative IRM overlay images of control and FACE1-depleted 35F VSMCs at t = 0 min (green) and t = 20 min (red); (**E**) Graph shows focal adhesion assembly over a 20-min period and represents the combined data of 15–20 cells per group pooled from three independent experiments (** *p* ≤ 0.01).

We next investigated whether prelamin A accumulation was specifically accountable for these changes in FA organisation. IF revealed that FAs were abundantly present throughout control VSMCs however, FACE1-depleted VSMCs displayed fewer focal adhesions (Figure 4A,B). Furthermore, similar to presenescent VSMCs, FAs in FACE1-depleted VSMCs were also more spherical than those of control VSMCs (Figure 4C). IRM showed that FACE1-depleted VSMCs also display increased FA dynamics compared to control VSMCs (Figure 4D,E). Collectively, this evidence suggests that prelamin A accumulation enhances FA dynamics during VSMC ageing.

### 3.3. VSMC Ageing and Prelamin A Accumulation Stimulate Migrational Persistence

**Figure 5:**
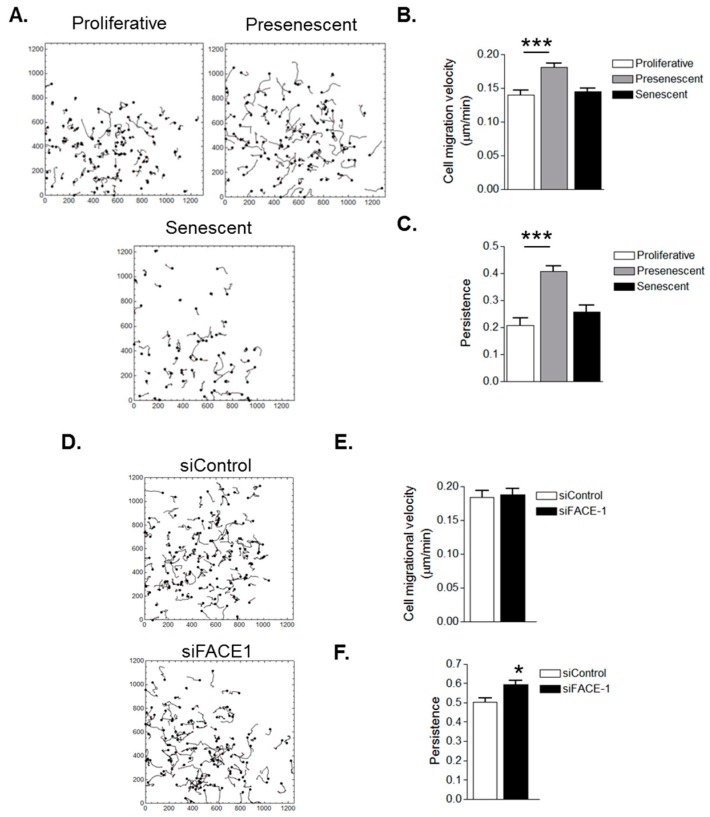
In Vitro ageing and prelamin A accumulation stimulate VSMC migrational persistence. (**A**) Analysis of randomly migrating single proliferative, presenescent and senescent VSMCs; Graphs show (**B**) migrational velocity and (**C**) persistence of proliferative, presenescent and senescent VSMCs. Data are based on the analysis of 61 (senescent) and 108 (proliferative and presenescent) individually tracked cells pooled from three independent experiments (*** *p* ≤ 0.0001); (**D**) Analysis of randomly migrating single control and FACE1-depleted 35F VSMCs; Graphs show (**E**) migrational velocity and (**F**) persistence of control and FACE1-depleted VSMCS. Data are based on the analysis of 121 (control) and 118 (FACE1-depleted) individually tracked cells pooled from three independent experiments (* *p* ≤ 0.05).

As FA dynamicity is indicative of a cell’s migratory capacity, we next used video time-lapse microscopy to capture random single cell migration and characterise the effect of ageing on VSMC migration. Presenescent VSMCs possessed increased migrational velocity compared to proliferative and senescent VSMCs (Figure 5A,B). Presenescent VSMC migration was also more directionally persistent than that of proliferative and senescent VSMCs (Figure 5A,C). These findings led us to test whether prelamin A accumulation specifically influenced cell migration. FACE1-depleted VSMCs exhibited no change to migrational velocity when compared with control VSMCs (Figure 5D,E). However, similar to presenescent VSMCs, FACE1-depleted VSMCs possessed enhanced migratory persistence compared with control cells (Figure 5D,F), confirming that prelamin A accumulation stimulates migrational persistence during VSMC ageing.

### 3.4. Prelamin A Accumulation Attenuates Rac1 Activity during VSMC Ageing

**Figure 6:**
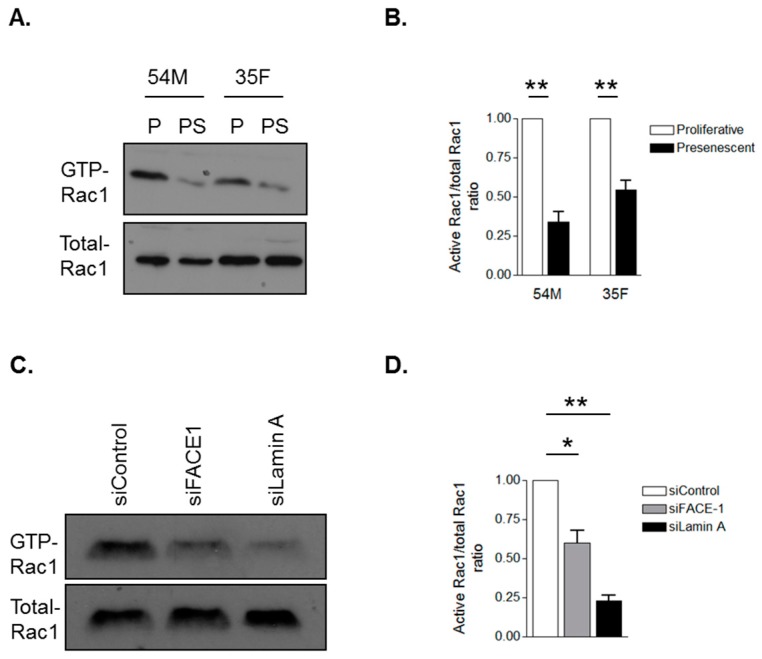
Prelamin A accumulation attenuates Rac1 activity. Glutathione-S-transferase (GST) pull-down assay using the GST-p21-associated protein-p21 Rho binding domain to precipitate GTP-bound Rac1 from (**A**) proliferative and presenescent 54M and 35F VSMCs and (**C**) control, FACE1- and lamin A/C-depleted VSMCs. Graphs show densitometry measurements of Active-Rac1/total-Rac1 ratios of (**B**) proliferative and presenescent VSMCs and (**D**) control, FACE-1- and lamin A/C-depleted VSMCs derived from three independent experiments (* *p* ≤ 0.05 and ** *p* ≤ 0.01).

Diminished activity of the small GTPase Rac1 has previously been demonstrated to promote migrational persistence, therefore, we investigated whether VSMC ageing and prelamin A accumulation alter Rac1 activity by performing GST pull-down experiments using the PAK-p21-binding domain that interacts with GTP-Rac1. WB revealed that Rac1 activity was attenuated in presenescent (Figure 6A,B) and FACE1-depleted (Figure 6C,D) VSMCs compared to their proliferative and control VSMCs respectively. To test whether prelamin A may be acting by interfering with lamin A/C function, we depleted lamin A/C in VSMCs. GST-pull down analysis revealed that Rac1 activity was attenuated in lamin A/C-depleted VSMCs compared to control VSMCs (Figure 6C,D). Together, this evidence suggests that prelamin A accumulation interferes with lamin A/C function to stimulate VSMC migrational persistence by suppressing Rac1 activity.

## 4. Discussion

The actin cytoskeleton regulates numerous cellular processes, including contractility, motility and proliferation, and age-related alterations to cytoskeletal organisation are detrimental to general VSMC functioning and cell fate [34,35]. Lamin A/C is indirectly coupled to F-actin via the LINC complex and disruption of either lamin A/C or the LINC complex induces cytoskeletal reorganisation and changes in cell motility [11,13,36,37]. VSMC ageing is driven by prelamin A accumulation and we show that presenescent VSMCs display changes in morphology that are indicative of cytoskeletal reorganisation [27]. FACE1-depletion induced prelamin A accumulation in proliferative VSMCs and recapitulated the elongated spindle-like morphology displayed by presenescent VSMCs. We have previously shown that presenescent and FACE1-depleted VSMCs stain positive for Ki67, suggesting that morphology changes induced by prelamin A are not due to cells exiting the cell cycle [27]. In addition to the morphology changes, we also show that prelamin A accumulation stimulated intrinsic migrational persistence of presenescent and FACE1-depleted VSMCs.

Importantly, we show for the first time that prelamin A accumulation attenuates activity of the small GTPase Rac1. Previous studies have demonstrated that Rac1 activity serves as a switch between random and directionally persistent migration and reduced Rac1 activity promotes migrational persistence in a variety of cell types [9]. This suggests that prelamin A accumulation attenuates Rac1 activity to promote migrational persistence during VSMC ageing. However, as prelamin A levels continue to increase and VSMCs enter senescence, migrational persistence subsides, suggesting that the impact of prelamin A on VSMC migration is dose dependent. Although further experimentation is required to elucidate the mechanism of how prelamin A accumulation influences Rac1 activity, we show that VSMCs depleted of lamin A/C also displayed reduced Rac1 activity, suggesting that prelamin A accumulation interferes with lamin A/C function at the NE. Lamin A/C is critical for nuclear organisation and provides structural support to the NE [38,39]. Progerin accumulation has been shown to disrupt nuclear organisation/mechanics in HGPS-derived fibroblasts and potentially these lamin A/C functions are also impaired by prelamin A accumulation during VSMC ageing [40]. In agreement with this notion, we have previously shown that nuclear morphology and heterochromatin alterations in aged VSMCs mimic those observed in HGPS-derived fibroblast cells [27]. Moreover, as integrin disruption has also been shown to disrupt Rac1 activity and induce migrational persistence, it is easy to envisage a mechanism whereby the LINC complex relays biophysical signals, derived from changes in nuclear mechanics, to F-actin and FAs to regulate Rac1 activity [9]. In agreement with this notion, we show that prelamin A accumulation triggered mislocalisation of nesprin-2 from the nuclear envelope in VSMCs. Clearly, further experimentation is now required to investigate the role of the LINC complex in regulating Rac1 activity and migrational persistence.

### Changes in Migrational Persistence during VSMC Ageing

In this current study, we analysed the migrational capacity of the three distinct VSMC growth phases termed proliferative, presenescence and senescence, using healthy VSMCs derived from a 35-year-old and 54-year-old donor. We have previously shown that VSMCs isolated from 70+ year old donors possess less proliferative capacity and are presenescent in tissue culture [27]. Importantly, a significant population of VSMCs in aortae of aged (70+ years) donors display prelamin A accumulation, stain positive for Ki67 and negative for senescence associated β-galactosidase activity, confirming that presenescent VSMCs exist in vivo [27]. In this current study, we show that single proliferative VSMCs were relatively slow and lacked migratory persistence which fits with earlier studies illustrating that VSMCs are slow-moving cells that exhibit random migration patterns [41,42]. Lack of migratory persistence suggests that young, healthy VSMCs within the vessel wall are dispersive with an explorative nature. Interestingly, presenescent VSMCs migrated more rapidly and persistently, indicative of more efficient directional migration. Ultimately, when VSMCs reached senescence, migration rates slowed. The inability of senescent VSMCs to conduct their migratory functions within the vessel may be detrimental to vascular health. Normally, VSMC migration is fundamental for vessel repair and VSMCs are a prominent cell-type at atherosclerotic sites where they aid the formation of a protective cap to stabilise rupture-prone lesions [42,43,44]. Increased VSMC migration, coupled with enhanced ECM deposition drives vessel remodeling and intimal thickening, which are major pathogenic factors in atherosclerotic plaque development [45,46,47]. We hypothesise that the enhancement of VSMC migration is primarily a protective mechanism allowing presenescent VSMCs a final attempt to repair vessel damage with a migratory ‘boost’ before they become senescent. Ultimately, when VSMCs reach senescence, migration and proliferation rates diminish, resulting in reduced recruitment of VSMCs to sites of vascular injury.

## References

[1] Wissler R.W., Robert L. (1996). Aging and cardiovascular disease: A summary of the Eighth Munster International Arteriosclerosis Symposium. Circulation.

[2] Lakatta E.G. (2003). Arterial and cardiac aging: Major shareholders in cardiovascular disease enterprises: Part III: Cellular and molecular clues to heart and arterial aging. Circulation.

[3] Tahir H., Niculescu I., Bona-Casas C., Merks R.M., Hoekstra A.G. (2015). An in silico study on the role of smooth muscle cell migration in neointimal formation after coronary stenting. J. R. Soc. Interface.

[4] Cai Y., Nagel D.J., Zhou Q., Cygnar K.D., Zhao H., Li F., Pi X., Knight P.A., Yan C. (2015). Role of cAMP-phosphodiesterase 1C signaling in regulating growth factor receptor stability, vascular smooth muscle cell growth, migration, and neointimal hyperplasia. Circ. Res..

[5] Shi N., Chen S.Y. (2015). Smooth Muscle Cell Differentiation: Model Systems, Regulatory Mechanisms, and Vascular Diseases. J. Cell Physiol..

[6] Fletcher D.A., Mullins R.D. (2010). Cell mechanics and the cytoskeleton. Nature.

[7] Hall A., Nobes C.D. (2000). Rho GTPases: Molecular switches that control the organization and dynamics of the actin cytoskeleton. Philos. Trans. R. Soc. Lond. B Biol. Sci..

[8] Fabbiano S., Menacho-Marquez M., Sevilla M.A., Albarran-Juarez J., Zheng Y., Offermanns S., Montero M.J., Bustelo X.R. (2014). Genetic dissection of the vav2-rac1 signaling axis in vascular smooth muscle cells. Mol. Cell Biol..

[9] Pankov R., Endo Y., Even-Ram S., Araki M., Clark K., Cukierman E., Matsumoto K., Yamada K.M. (2005). A Rac switch regulates random versus directionally persistent cell migration. J. Cell Biol..

[10] Chambliss A.B., Khatau S.B., Erdenberger N., Robinson D.K., Hodzic D., Longmore G.D., Wirtz D. (2013). The LINC-anchored actin cap connects the extracellular milieu to the nucleus for ultrafast mechanotransduction. Sci. Rep..

[11] Crisp M., Liu Q., Roux K., Rattner J.B., Shanahan C., Burke B., Stahl P.D., Hodzic D. (2006). Coupling of the nucleus and cytoplasm: Role of the LINC complex. J. Cell Biol..

[12] Guilluy C., Osborne L.D., Van Landeghem L., Sharek L., Superfine R., Garcia-Mata R., Burridge K. (2014). Isolated nuclei adapt to force and reveal a mechanotransduction pathway in the nucleus. Nat. Cell Biol..

[13] Lombardi M.L., Jaalouk D.E., Shanahan C.M., Burke B., Roux K.J., Lammerding J. (2011). The interaction between nesprins and sun proteins at the nuclear envelope is critical for force transmission between the nucleus and cytoskeleton. J. Biol. Chem..

[14] Young S.G., Fong L.G., Michaelis S. (2005). Prelamin A, Zmpste24, misshapen cell nuclei, and progeria—New evidence suggesting that protein farnesylation could be important for disease pathogenesis. J. Lipid. Res..

[15] Rusinol A.E., Sinensky M.S. (2006). Farnesylated lamins, progeroid syndromes and farnesyl transferase inhibitors. J. Cell Sci..

[16] Corrigan D.P., Kuszczak D., Rusinol A.E., Thewke D.P., Hrycyna C.A., Michaelis S., Sinensky M.S. (2005). Prelamin A endoproteolytic processing in vitro by recombinant Zmpste24. Biochem. J..

[17] Kudlow B.A., Kennedy B.K., Monnat R.J. (2007). Werner and Hutchinson-Gilford progeria syndromes: Mechanistic basis of human progeroid diseases. Nat. Rev. Mol. Cell Biol..

[18] Goldman R.D., Shumaker D.K., Erdos M.R., Eriksson M., Goldman A.E., Gordon L.B., Gruenbaum Y., Khuon S., Mendez M., Varga R. (2004). Accumulation of mutant lamin A causes progressive changes in nuclear architecture in Hutchinson-Gilford progeria syndrome. Proc. Natl. Acad. Sci. USA.

[19] Scaffidi P., Misteli T. (2005). Reversal of the cellular phenotype in the premature aging disease Hutchinson-Gilford progeria syndrome. Nat. Med..

[20] Huang S., Risques R.A., Martin G.M., Rabinovitch P.S., Oshima J. (2008). Accelerated telomere shortening and replicative senescence in human fibroblasts overexpressing mutant and wild-type lamin A. Exp. Cell Res..

[21] Eriksson M., Brown W.T., Gordon L.B., Glynn M.W., Singer J., Scott L., Erdos M.R., Robbins C.M., Moses T.Y., Berglund P. (2003). Recurrent de novo point mutations in lamin A cause Hutchinson-Gilford progeria syndrome. Nature.

[22] Gruenbaum Y., Margalit A., Goldman R.D., Shumaker D.K., Wilson K.L. (2005). The nuclear lamina comes of age. Nat. Rev. Mol. Cell Biol..

[23] Kutscheidt S., Zhu R., Antoku S., Luxton G.W., Stagljar I., Fackler O.T., Gundersen G.G. (2014). FHOD1 interaction with nesprin-2G mediates TAN line formation and nuclear movement. Nat. Cell Biol..

[24] Dechat T., Pfleghaar K., Sengupta K., Shimi T., Shumaker D.K., Solimando L., Goldman R.D. (2008). Nuclear lamins: Major factors in the structural organization and function of the nucleus and chromatin. Genes Dev..

[25] Merideth M.A., Gordon L.B., Clauss S., Sachdev V., Smith A.C., Perry M.B., Brewer C.C., Zalewski C., Kim H.J., Solomon B. (2008). Phenotype and course of Hutchinson-Gilford progeria syndrome. N. Engl. J. Med..

[26] Bergo M.O., Gavino B., Ross J., Schmidt W.K., Hong C., Kendall L.V., Mohr A., Meta M., Genant H., Jiang Y. (2002). Zmpste24 deficiency in mice causes spontaneous bone fractures, muscle weakness, and a prelamin A processing defect. Proc. Natl. Acad. Sci. USA.

[27] Ragnauth C.D., Warren D.T., Liu Y., McNair R., Tajsic T., Figg N., Shroff R., Skepper J., Shanahan C.M. (2010). Prelamin A acts to accelerate smooth muscle cell senescence and is a novel biomarker of human vascular aging. Circulation.

[28] Warren D.T., Tajsic T., Porter L.J., Minaisah R.M., Cobb A., Jacob A., Rajgor D., Zhang Q.P., Shanahan C.M. (2015). Nesprin-2-dependent ERK1/2 compartmentalisation regulates the DNA damage response in vascular smooth muscle cell ageing. Cell Death Differ..

[29] Pacheco L.M., Gomez L.A., Dias J., Ziebarth N.M., Howard G.A., Schiller P.C. (2014). Progerin expression disrupts critical adult stem cell functions involved in tissue repair. Aging (Albany NY).

[30] Booth-Gauthier E.A., Du V., Ghibaudo M., Rape A.D., Dahl K.N., Ladoux B. (2013). Hutchinson-Gilford progeria syndrome alters nuclear shape and reduces cell motility in three dimensional model substrates. Integr. Biol..

[31] Proudfoot D., Shanahan C. (2012). Human vascular smooth muscle cell culture. Methods Mol. Biol..

[32] Zhang Q., Minaisah R.M., Ferraro E., Li C., Porter L.J., Zhou C., Gao F., Zhang J., Rajgor D., Autore F. (2016). N-terminal nesprin-2 variants regulate beta-catenin signalling. Exp. Cell Res..

[33] Holt M.R., Calle Y., Sutton D.H., Critchley D.R., Jones G.E., Dunn G.A. (2008). Quantifying cell-matrix adhesion dynamics in living cells using interference reflection microscopy. J. Microsc..

[34] Hall A. (1998). Rho GTPases and the actin cytoskeleton. Science.

[35] Mayanagi T., Sobue K. (2011). Diversification of caldesmon-linked actin cytoskeleton in cell motility. Cell Adhes. Migr..

[36] Haque F., Lloyd D.J., Smallwood D.T., Dent C.L., Shanahan C.M., Fry A.M., Trembath R.C., Shackleton S. (2006). SUN1 interacts with nuclear lamin A and cytoplasmic nesprins to provide a physical connection between the nuclear lamina and the cytoskeleton. Mol. Cell Biol..

[37] Khatau S.B., Kusuma S., Hanjaya-Putra D., Mali P., Cheng L., Lee J.S., Gerecht S., Wirtz D. (2012). The differential formation of the LINC-mediated perinuclear actin cap in pluripotent and somatic cells. PLoS ONE.

[38] Lammerding J., Schulze P.C., Takahashi T., Kozlov S., Sullivan T., Kamm R.D., Stewart C.L., Lee R.T. (2004). Lamin A/C deficiency causes defective nuclear mechanics and mechanotransduction. J. Clin. Investig..

[39] Swift J., Ivanovska I.L., Buxboim A., Harada T., Dingal P.C., Pinter J., Pajerowski J.D., Spinler K.R., Shin J.W., Tewari M. (2013). Nuclear lamin-A scales with tissue stiffness and enhances matrix-directed differentiation. Science.

[40] Verstraeten V.L., Ji J.Y., Cummings K.S., Lee R.T., Lammerding J. (2008). Increased mechanosensitivity and nuclear stiffness in Hutchinson-Gilford progeria cells: Effects of farnesyltransferase inhibitors. Aging Cell.

[41] Rocnik E.F., Chan B.M., Pickering J.G. (1998). Evidence for a role of collagen synthesis in arterial smooth muscle cell migration. J. Clin. Investig..

[42] Li S., Chow L.H., Pickering J.G. (2000). Cell surface-bound collagenase-1 and focal substrate degradation stimulate the rear release of motile vascular smooth muscle cells. J. Biol. Chem..

[43] Dickson B.C., Gotlieb A.I. (2003). Towards understanding acute destabilization of vulnerable atherosclerotic plaques. Cardiovasc. Pathol..

[44] Naghavi M., Libby P., Falk E., Casscells S.W., Litovsky S., Rumberger J., Badimon J.J., Stefanadis C., Moreno P., Pasterkamp G. (2003). From vulnerable plaque to vulnerable patient: A call for new definitions and risk assessment strategies: Part II. Circulation.

[45] Izzo J.L., Shykoff B.E. (2001). Arterial stiffness: Clinical relevance, measurement, and treatment. Rev. Cardiovasc. Med..

[46] Orlandi A., Marcellini M., Spagnoli L.G. (2000). Aging influences development and progression of early aortic atherosclerotic lesions in cholesterol-fed rabbits. Arterioscler. Thromb. Vasc. Biol..

[47] Louis S.F., Zahradka P. (2010). Vascular smooth muscle cell motility: From migration to invasion. Exp. Clin. Cardiol..

